# Ultra-processed food consumption in the central division of Fiji

**DOI:** 10.1186/s12916-025-03947-w

**Published:** 2025-02-21

**Authors:** Aliyah Palu, Joseph Alvin Santos, Daisy Coyle, Maria Shahid, Juliette Crowther, Gade Waqa, Colin Bell, Jacqui Webster, Briar McKenzie

**Affiliations:** 1 The George Institute for Global Health, University of New South Wales, Sydney, NSW 2042 Australia; 2 Department of Business Economics, Health, and Social Care (DEASS), University of Applied Sciences and Arts of Southern Switzerland (SUPSI), Manno, Switzerland; 3 Pacific Research Centre for the Prevention of Obesity and Non-Communicable Diseases, Fiji National University, Tamavua Campus, Suva, Fiji; 4 Institute for Health Transformation, Deakin University, Geelong, Australia

**Keywords:** Ultra-processed foods, Fiji, Small Island Developing States, Non-communicable diseases

## Abstract

**Background:**

Processed packaged foods are readily available in Fiji; however, the extent to which ultra-processed foods (UPFs) currently contribute to energy and nutrient intake is unknown. This study aimed to assess the contribution of UPFs to total energy intake and nutrients of concern (sodium, sugar, fat) in a representative sample of adults in the central division of Fiji, identify the main food category sources of UPFs and assess variation by sociodemographic characteristics.

**Methods:**

A random sample of 700 adults was selected from two statistical enumeration areas (one semi-urban, one rural). Participant characteristics were collected, and a three-pass 24-h diet recall was undertaken. Foods consumed were coded based on the level of processing, in alignment with the NOVA categorisation system.

**Results:**

The contribution of UPFs to total energy, fat, sugar, and sodium intake and dietary sources of UPFs (based on the per cent daily energy contribution of UPFs from food groups) were estimated and assessed by sex, age group, ethnicity and location. A total of 534 adults participated (76% response rate, 50% female). UPFs contributed 21.5% (95% CI, 21.4% to 26.6%) of total energy intake, 22.8% (95% CI 20.5% to 25.1%) of total sodium intake, 24.0% (95% CI, 21.4% to 26.6%) of sugar intake and 18.6% (95% CI 16.5% to 20.7%) of total fat intake. Key food group contributors to UPF intake were bread and bakery products 42.9% (38.3% to 47.6%), non-alcoholic beverages 26.8% (22.4% to 31.1%), convenience foods 8.6% (6.3% to 10.8%), and meat, poultry, and meat alternatives 6.9% (4.8% to 8.9%). The contribution of UPFs to sodium, sugar and fat intake was similar for men and women; however, differences were observed by age group, ethnicity and region (semi-urban compared to rural).

**Conclusions:**

This study identified that UPFs appear to be a large contributor to energy, sodium, fat and sugar intake in adults in the Central division of Fiji. A reduction of UPF consumption in Fiji may lead to a reduction of harmful nutrients such as sodium, fat, and sugar, crucial to reducing the diet-related burden of disease.

**Supplementary Information:**

The online version contains supplementary material available at 10.1186/s12916-025-03947-w.

## What is already known?

Pacific Island countries experience a high prevalence of non-communicable diseases (NCDs) due to an ongoing nutrition transition away from traditional foods and increased accessibility and affordability of imported pre-packaged foods high in sodium, sugar and fat. Ultra-processed foods (UPFs) are food items that often consist of higher at-risk nutrients, have undergone higher levels of food processing and have been directly associated with higher risk of mortality and NCDs.

## What this study adds

This is the first study to quantitatively assess UPF consumption in Fiji. This cross-sectional survey of over 500 adults in the central division of Fiji, found that UPFs contributed around a fifth of total energy, sodium and sugar intake and total fat intake. Further, UPFs mainly came from bread and bakery products, non-alcoholic beverages and convenience foods. These findings suggest that interventions targeting UPFs could support Fijians to reduce their consumption of sodium, sugar and fat.

## Background

In the Western Pacific region, non-communicable diseases (NCDs) such as heart disease, stroke, diabetes and cancer account for nearly nine in 10 deaths [1] and are considered an epidemic. In Fiji, the prevalence of cardiovascular disease, cancers and chronic respiratory diseases (such as asthma) is high, contributing to more than 85% of deaths annually [2]. Unhealthy diets are a major risk factor for NCDs [3, 4]. Fiji has undergone a nutrition transition from traditional diets consisting of root crop staples, fruits and seafood to diets based on imported packaged foods [5]. It has been established that such foods are contributing to the high intake of sodium and sugar in Fiji [6]. Consequently, intakes of sugar and sodium in the country exceed the World Health Organization (WHO) guidelines by two and three times, respectively [7].

The level of processing of different foods is increasingly recognised as a factor that contributes to the burden of diet-related non-communicable diseases [8, 9]. However, food processing categorisation and definitions have been heavily scrutinised suggesting differing perspectives on how food items should be categorised [5]. In 2017, a systematic review on processed foods and its impact on health outcomes reported that processed foods are generally known to be high in at-risk nutrients (sodium, sugar and fats) as well as being energy dense and lack ‘positive’ nutrients such as fibre, protein, vitamins and minerals [10]. Foods that undergo further processing are considered Ultra-processed Foods (UPFs), which include breakfast cereals, savoury snacks and SSB [11]. A recent systematic review has indicated that increased exposure to UPFs is associated with a higher risk of all-cause mortality, heart disease-related mortality, type 2 diabetes and obesity [12]. UPFs are generally defined using the NOVA classification system, which classifies food products into four categories [13]. These groups include; Group 1: Unprocessed and minimally processed foods, Group 2: Processed culinary ingredients, Group 3: Processed foods and Group 4: UPFs [13]. UPFs contribute to more than half of the total dietary energy intake in countries such as the USA, Canada, and the UK [13]. UPFs tend to be high in energy and low in nutritional quality, often containing high levels of free sugars, saturated and trans fats and sodium [14]. Associations have been identified between higher UPF consumption and an increased risk of diabetes, obesity, hypertension, stroke, coronary heart disease (CHD), along with certain types of cancers [15].

The contribution of UPFs to diets in Fiji is currently unknown, although likely to be increasing, given the increased importation of packaged foods into the region [16]. Therefore, the aim of this study was to assess the contribution of UPFs to total energy intake and nutrients of concern (sodium, sugar, fat) in a representative sample of adults in the central division of Fiji, along with identifying the main food category sources of UPFs. A secondary aim was to assess socio-demographic variation in intakes of UPF.

## Methods

This study is part of a larger Global Alliance for Chronic Diseases (GACD) project to monitor changes in diet and support the scale-up of food policy interventions [7, 17]. The project received ethics clearance from the University of New South Wales (#HC200469) and Fiji National University College Human Health Research Ethical Committee (CHREC264.20).

### Sample size and recruitment

Waidamudamu Medical Zone (urban) and Deuba Medical Zone (rural) were randomly selected from enumeration areas in the Central Division of Fiji. A household listing was conducted from October to December 2021, as the most recent census data in 2017 was unavailable at the time of this study. Basic demographic data was collected from individuals over the age of 18 from each household. This data was then used to select a proportionate stratified sample by demographics (age, sex and ethnicity). Further information on the household listing has been previously published [7]. The sample size for this study was calculated for the overarching survey aims and objectives [7].

Between March and July 2022, trained research assistants visited the houses of selected participants to inform them of the study and invited people to participate. Participant information sheets and consent forms were available in English, Hindi, and Fijian. These translated information sheets were certified by senior Fijian researchers at Fiji National University who checked the translated information by translating back to English. Surveys took place in the participant’s home or at another convenient place and spanned approximately an hour. Research assistants went through each stage of the survey with participants and collected information electronically on handheld tablets; this included information on participant demographics (gender, age, ethnicity, and location) and the 24-h diet recall survey. Further information on survey data collection has been previously published [7, 17].

### Twenty-four-hour diet recall

This study collected dietary data using 24-h recalls. The Intake24 diet recall application (hereafter Intake24) was used along with food composition data from the UK and New Zealand [18]. Intake24 was adapted for Fiji by adding one hundred commonly consumed foods. These foods were identified using information from the 2014/2015 Fiji National Nutrition Survey [19] and in discussion with dietitians at the Ministry of Health, Fiji. If a direct match to food items in the existing UK or New Zealand database was not possible, food items were matched to items already within the database based on being within 25% of key nutrient components [20]. Interviewers went through Intake24 with participants, guiding the multiple pass recall process. The diet recall process recorded what participants ate at specific times (e.g. breakfast, snacks, lunch, and dinner). Research assistants also guided participants with prompts to further understand portion sizes of foods consumed and the preparation of foods and brands of foods (where appropriate). Further information on the use of the Intake24 application has been previously published [7].

### Data processing

Energy and nutrients of concern (sugar, sodium and fat) intakes were calculated as kilojoules per day for energy, mg per day for sodium and grams per day for sugar and fat. Fat and sugar intake were reported as a percentage of total energy intake [7, 21].

Based on previous surveys conducted in Fiji [22, 23] we categorised foods reported in the 24-h diet recall into 17 categories (1—alcohol, 2—bread and bakery products, 3—cereal and grain products, 4—coconut products, 5—confectionery, 6—convenience foods (including takeaway meals ready meals, meal kits, pre-prepared salads and sandwiches), 7—dairy, 8—edible oil and oil emulsions, 9—egg and egg products, 10—fruit, vegetables, nuts and legumes, 11—meat, poultry and meat alternatives, 12—mixed cooked dishes, 13—non-alcoholic beverages, 14—sauces, dressings, spreads and dips, 15—seafood and seafood products, 16—snack foods (sweet and savoury snacks), and 17—table sugars, honey and related products (such as syrups and molasses)).

### NOVA categorisation

The NOVA classification system was used to categorise individually reported foods by level of processing [13]. Group 1 food products were defined as unprocessed food ingredients, considered natural foods that are edible parts of plants or animal products such as eggs and milk. Group 2 food products were defined as processed culinary ingredients such as butter and oils. Group 3 food products were defined as products that have undergone preservation cooking processes, for example, canned fruits. Group 4 food products were defined as any foods that have been made using a variety of industrial techniques and processes for example, sugar-sweetened beverages (SSBs). For this study, unprocessed and minimally processed foods (Group 1) and processed culinary ingredients (Group 2) were combined due to Group 1 and 2 often being combined and used to prepare Group 3 and Group 4. Following a previously published study [24], food products such as mixed cooked dishes/dishes cooked at home were adapted to a NOVA classification. A list of assumptions is described in Table 1.

**Table 1:** NOVA classification assumptions for food products assigned to NOVA groups

Food products	NOVA classification assumption
Mixed cooked dishes	• Foods that were considered ‘mixed cooked dishes’ were coded to NOVA according to the level of processing of the main ingredient [24]. Examples of ‘mixed cooked dishes’ included any stir-fry, hot pot, curry, chicken and sweetcorn soup, chicken casseroles and stews, chop suey and fried rice. For example, a ‘mixed cooked dish’ such as chicken stir fry, was classified as processed, due to the level of processing of products added to the dish • ‘Mixed cooked dishes’ such as chicken pizza, fried chicken, and burgers were categorised as ultra-processed, due to the higher level of processing of the main ingredient and overall dish
Bread and bakery products	• In line with prior research that found bread and bakery products are often purchased [7], we assumed that products such as bread rolls and loaves of bread, along with products like muffins and biscuits, were likely store-bought (unless explicitly stated by participants that they were homemade) and therefore were categorised as ultra-processed • We assumed that Roti was mostly homemade and therefore categorised as processed. We also assumed that other breads such as ‘bara birth’, ‘plain naan bread’, ‘chapati’, and ‘pitta’ were homemade and were categorised as processed
Coffee	• Coffee that was reported as ‘ready to drink’, ‘Coffee Mate’ or ‘instant coffee’ was categorised as ultra-processed • Juices that were not explicitly recorded as ‘freshly squeezed’ were also categorised as ultra-processed [24] • We assumed that juices that were recorded as ‘mixed fruit drinks, ready to drink’ were ultra-processed • Vitamin water and lemon tea (which is often homemade) were categorised as processed

### Statistical analysis

Energy intake from the foods and beverages contributing to each NOVA classification was calculated and computed as a percentage of total energy intake (%) from UPF. This was calculated per person, with averages then reported across the sample and predefined subgroups (gender, age, ethnicity and location). This formula was also applied for sodium, sugar, and fat intake. The main dietary sources of UPFs were calculated by estimating the contribution of each food category to total daily energy intake across each NOVA classification. Analyses were weighted to reflect the probability of individual selection (sample weight) and to match the population of Deuba and Waidamudamu (population weight). Weights were based on predefined participant subgroups such as sex (women, men), age group (18–44 and 45–85), ethnicity (iTaukei (Indigenous Fijian) and Fijian of Indian descent or other) and area (Deuba and Waidamudamu). Results were reported as mean proportion with standard error or 95% confidence interval (CI). Survey data was analysed using STATA BE V17.0 for Windows (Stata Corp LP, College Station, TX, USA).

## Results

### Population characteristics

Overall, 534 people participated in the survey (response rate: 76%), of which 50.4% were women (*n* = 272). Over 60% of the population were aged 18 to 44 years and approximately half of the population were Itaukei (46.8%). Most participants were from Deuba (60%) and had attained a secondary education (69.4%). Characteristics of the study population have been published previously [7].

### Contribution of ultra-processed foods to energy and nutrient intake

In total, 5136 food items were reported in the 24-h dietary recall by participants, 67.4% were unprocessed or minimally processed, 17.4% were processed and 15.2% were UPF. Of the 5136 food items, a total of 565 different food items were reported and of these, 169 products were classified as ultra-processed. Overall, UPFs contributed to 21.5% (95% CI 21.4 to 26.6) of total energy intake, 22.8% (95% CI 20.5 to 25.1) of sodium intake, 24.0% (95% CI 21.4 to 26.6) of sugar intake, and 18.6% (95% CI to 16.5 to 20.7) of fat intake. UPF consumption was similar for men and women, however, higher UPF consumption was found for Itaukei (26.8% (95% CI 23.8 to 29.8)) than for Fijians of Indian descent and other ethnicities (16.8% (95% CI 14.2 to 19.3)). UPFs contributed more to energy intake for individuals living in Deuba at 23.4% (95% CI 20.6 to 26.1) than those from Waidamudamu at 18.5% (95% CI 16.1 to 21.0) (Table 2).

**Table 2:** Ultra-processed foods contribution to energy, sodium, sugar and fat intake (mean % and 95% CI), for the total sample (*n* = 534) and by sociodemographic characteristics

		**By sex**		**By age group**		**By ethnicity**		**By area**	
	**Total**	**Female**	**Male**	**18 to 44 years**	** ≥ 45 years**	**Itaukei**	**FID and FOD**	**Deuba**	**Waidamudamu**
Energy	21.5 (19.5 to 23.4)	21.9 (19.2 to 24.6)	21.0 (18.3 to 23.7)	22.3 (19.8 to 24.9)	19.9 (17.1 to 22.8)	26.8 (23.8 to 29.8)^b^	16.8 (14.2 to 19.3)^b^	23.4 (20.6 to 26.1)^c^	18.5 (16.1 to 21.0)^c^
Sodium	22.8 (20.5 to 25.1)	23.6 (20.4 to 26.8)	22.0 (18.8 to 25.3)	22.9 (19.9 to 25.8)	22.6 (19.0 to 26.3)	30.8 (27.0 to 34.7)^b^	15.8 (13.1 to 18.4)^b^	24.5 (21.3 to 27.7)	20.2 (17.0 to 23.3)
Sugar	24.0 (21.4 to 26.6)	23.8 (20.2 to 27.4)	24.2 (20.5 to 27.9)	26.4 (22.9 to 30.0)^a^	19.8 (16.3 to 23.3)^a^	24.7 (21.0 to 28.4)	23.4 (19.7 to 27.0)	24.8 (21.1 to 28.5)	22.8 (19.4 to 26.2)
Fat	18.6 (16.5 to 20.7)	19.4 (16.5 to 22.4)	17.7 (14.7 to 20.8)	18.7 (15.9 to 21.4)	18.5 (15.1 to 21.8)	24.3 (20.9 to 27.7)^b^	13.6 (11.0 to 16.2)^b^	20.6 (17.6 to 23.6)^c^	15.5 (12.8 to 18.2)^c^

*FID and FOD*, Fijian Indian and Fijian Other Descent; 0.05 significance level

^a^Significant difference by age

^b^Significant difference by ethnicity

^c^Significant difference by area

The contribution of UPFs to sodium, sugar and fat intake was similar for men and women. However, sugar intake from UPFs was higher for those aged 18 to 44 years (26.4% (95% CI 22.9 to 30.0) in comparison to those 45 years and up (19.8% (95% CI 19.7 to 27.0). UPFs contributed to nearly double the sodium intake for Itaukei (30.8% (95% CI 27.0 to 34.7) compared to Fijian Indian descent and other ethnicities (15.8% (95% CI 13.1 to 18.4). Similarly, UPFs contributed to higher fat intake in Itaukei (24.3% (95% CI 20.9 to 27.7) than in FID and FOD (13.6% (95% CI 11.0 to 16.2) (see Table 2).

### Processed and unprocessed or minimally processed foods

Overall, unprocessed or minimally processed foods contributed 47.6% (95% CI 45.3 to 49.9) to energy intake (see Additional file 1: Table 1). In terms of the four nutrients, unprocessed or minimally processed foods contributed 39.1% (95% CI 36.4 to 41.7) to overall sodium intake, 52.9% (95% CI 50.2 to 55.7) to overall sugar intake, and 44.4% (95% CI 41.7 to 47.2) to overall fat intake (see Additional file 1: Table 1). The contribution of unprocessed or minimally processed foods to energy and sugar intake showed no significant differences across sociodemographic categories (see Additional file 1: Table 1). The contribution of unprocessed or minimally processed foods to sodium intake was similar for men and women across age groups 18–44 years and 45 years and up, and in Deuba and Waidamudamu (see Additional file 1: Table 1). The contribution of unprocessed or minimally processed and processed foods to fat intake showed no significant difference in men and women, age groups and area.

The contribution of processed foods to energy and sugar intake showed no significant differences across sociodemographic categories (see Additional file 1: Table 2). The contribution of processed foods to sodium intake was similar for men and women, across age groups 18 to 44 years and 45 years and up, and in Deuba and Waidamudamu (see Additional file 1: Table 2). However, there was a significant difference of processed foods contributing to sodium intake in Itaukei 31.2% (95% CI 27.1 to 35.2) compared to Fijian Indian and other descent 44.3% (95% CI 40.7 to 47.8). Furthermore, the contribution of processed foods to fat intake showed a significant difference in Itaukei 30.3% (95% CI 26.3 to 34.2) and Fijian Indian and Other descent 42.8% (95% CI 39.3 to 46.4) (see Additional file 1: Table 2). Processed foods contributed 30.9% (95% CI 28.8 to 33.1) to overall energy intake (see Additional file 1: Table 2). Across the four nutrients, processed foods contributed 38.2% (95% CI 35.5 to 40.8) to overall sodium intake, 23.1% (95% CI 20.9 to 25.2) to overall sugar intake and 37.0% (95% CI 34.3 to 39.6) to overall fat intake (see Additional file 1: Table 2).

### Sources of unprocessed or minimally processed, processed and ultra-processed foods

Bread and bakery products were the main source of UPFs (42.9%) followed by non-alcoholic beverages 26.8%, convenience foods 8.6%, and meat, poultry, and meat alternatives 6.9% (Table 3). Bread and bakery products were also the main source of processed foods (35.2%) mixed cooked dishes (25.5%) and fruit, vegetables, nuts and legumes (25.5%) were the main sources of unprocessed or minimally processed foods (25.5%).

**Table 3:** Percent of energy from food groups contributing to unprocessed or minimally processed, processed, and ultra-processed food categories

Food group	Unprocessed or minimally processed	Processed	Ultra-processed
Alcohol	0.0 (0.0 to 0.0)	0.3 (0.0 to 1.0)	0.7 (0.0 to 1.6)
Bread and bakery products	0.1 (0.0 to 0.2)	**35.2 (31.5 to 39.0)**	**42.9 (38.3 to 47.6)**
Cereal and grain products	21.8 (19.8 to 23.7)	6.9 (4.7 to 9.1)	1.7 (0.6 to 2.7)
Coconut products	0.2 (0.0 to 0.3)	2.8 (1.3 to 4.3)	0.0 (0.0 to 0.0)
Confectionery	0.0 (0.0 to 0.0)	0.0 (0.0 to 0.0)	0.6 (0.0 to 1.1)
Convenience foods	0.6 (0.0 to 1.4)	5.0 (3.1 to 6.9)	**8.6 (6.3 to 10.8)**
Dairy	1.6 (1.0 to 2.2)	1.3 (0.4 to 2.2)	2.3 (0.8 to 3.8)
Edible oil and oil emulsions	3.6 (2.6 to 4.6)	0.3 (0.0 to 0.7)	0.6 (0.2 to 1.0)
Egg and egg products	3.5 (2.6 to 4.5)	0.0 (0.0 to 0.0)	0.0 (0.0 to 0.1)
Fruit, vegetables, nuts, and legumes	25.5 (23.1 to 27.9)	0.4 (0.1 to 0.6)	0.4 (0.0 to 0.7)
Meat, poultry, and meat alternatives	2.7 (1.6 to 3.7)	**0.6 (0.2 to 1.1)**	**6.9 (4.8 to 8.9)**
Mixed cooked dishes	25.5 (23.1 to 27.8)	**25.9 (22.1 to 29.7)**	1.1 (0.0 to 2.3)
Non-alcoholic beverages	2.2 (1.2 to 3.2)	**11.6 (8.7 to 14.4)**	**26.8 (22.4 to 31.1)**
Sauces, dressings, spreads, and dips	0.2 (0.0 to 0.6)	1.4 (0.3 to 2.6)	2.7 (1.0 to 4.4)
Seafood and seafood products	0.5 (0.2 to 0.8)	8.2 (5.8 to 10.5)	2.6 (0.9 to 4.3)
Snack foods	0.0 (0.0 to 0.0)	0.0 (0.0 to 0.0)	1.9 (0.6 to 3.3)
Sugars, honey, and related products	12.0 (10.5 to 13.5)	0.0 (0.0 to 0.0)	0.3 (0.0 to 0.7)
Total	100	100	100

Bold numbers indicate significant values with 0.05 significance level

Unprocessed or minimally processed refers to products that have undergone minimal food processing methods or are culinary ingredients

## Discussion

This is the first study to quantitively assess UPF consumption in Fiji. Based on 24-h-recall data, UPFs contributed almost a fifth of total energy, sodium and sugar intake and 19% of total fat intake in a representative sample of adults in the Central Division of Fiji. UPFs mainly came from bread and bakery products, non-alcoholic beverages, and convenience foods. The contribution of UPFs to energy intake was similar by gender but was highest for those of iTaukei ethnicity, younger adults and people living in rural areas of Fiji. These findings suggest that interventions targeting UPFs may also help Fijians reduce their consumption of sodium, sugar and fat.

### Global context

In Mexico and Brazil, studies found that UPFs contribute to 30% [25] and 13–21% [26] of total energy intake, respectively. Our findings are similar to those in Brazil, suggesting that Fiji may be at a similar stage of the nutrition transition [27]. The finding that non-alcoholic beverages were a major contributor to UPF consumption in Fiji aligns with studies conducted on food imports, that have identified increasing SSB imports since 2000 [28]. Other studies conducted in low- and middle-income countries (LMICs) in Asia have shown an increase in sales of UPFs from 2000 to 2013, especially SSBs. The growth in sales of UPFs is likely due to increased marketing and availability of UPFs in these countries, driving demand for these foods and consequently shifting dietary patterns [29]. In higher income countries UPFs contribute a greater percentage of population energy intake. For example, in the USA (> 50% of energy intake) [30], UK (57% of energy intake in adults) [31, 32] and Australia (43.6% of energy intake) [26, 33]. Fiji has an opportunity to avoid such high UPF intakes by introducing regulations and promoting unprocessed or minimally processed foods such as mixed-cooked dishes and fruit and vegetables.

### Food policy recommendations and dietary guidelines to support reducing nutrients of concern

Consumption of sodium and sugar in Fiji exceeds recommendations by the WHO by at least twofold and threefold, respectively [7]. As such, reducing excess sodium and sugar intake in Fiji is a public health priority. The present study has identified that UPFs are key contributors to these nutrients, in addition to fat. In our previous work, we identified bread and bakery products are key contributors to sodium and sugar intake in a representative sample of adults in central Fiji, with people mainly purchasing these products from supermarkets or rural stores [7]. The present study builds on this work by examining the ingredient lists of packaged bread and bakery products (for example, those sold within supermarkets often contain additives that have an extended shelf life in comparison to freshly baked products). We have found that several items within the bread and bakery categories are classified as ultra-processed. Given that bread is a key contributor to energy intake overall, this means that bread is also the leading source of energy from UPFs in Fiji. However, there is some debate around the harms of specific food groups such as bread based on processing level. Cordova et al. investigated the health impacts of UPF consumption in a large prospective cohort study, finding that higher UPF consumption increased the risk of cancer and cardiometabolic multimorbidity, but they did not find a relationship between consumption of ultra-processed breads and cereals with disease risk specifically [34].

Nutrition education is a crucial opportunity to inform Pacific Islanders on healthy diets, food systems and food production [35]. Across the Pacific, there have been a variety of initiatives to support programmes that educate children on the importance of healthy diets and the higher prevalence of NCDs in the region [36]. For example, the Pacific School Food Network aims to improve the health of children, families and communities through healthier school food and nutrition environments [35]. However, there is little evidence that there is UPF nutrition education in the Pacific in terms of UPFs being a source of high-risk nutrients. Similarly, in Fiji, there are programmes such as The Health Schools Programme aimed at improving diet through the ‘no junk food policy’ [37]. This shows that there is an opportunity for specific UPF education to circumvent the negative health outcomes of over-consumption of UPFs. Non-alcoholic beverages continue to contribute to poor health in Fiji despite efforts to reduce consumption by taxing SSBs. Currently, Fiji’s SSB tax is 2.00/L FJD import excise duty, with a separate tax for locally produced SSB of 0.35/L FJD [38]. Our study indicates that there is an opportunity to increase this tax and impose similar taxes on other products. The level of processing could be used as a taxing metric following examples in Mexico and Columbia. In 2014, Mexico introduced an 8% sales tax on non-essential food products high in sodium, added sugars or solid fats [39]. Colombia has recently introduced a 10% tax that will increase over the next two years to 20% in 2025 for foods such as sausages, cereals, condiments, jellies and jams [39]. In the same way that Fiji have supported the SSB tax, there is an opportunity for the food science community in Fiji, to group together to advocate for UPFs to be taxed.

The availability, affordability, and desirability of UPFs have been increasing in the Pacific [40]. A study conducted in Vanuatu found that Vanuatu’s trade liberalisation increased the importation of UPFs and consequently increased their consumption [41]. Likewise, studies in Fiji have linked trade liberalisation to the increased importation of processed foods, which contributes to poor health outcomes [42]. Regulations to counter this trend and protect health are needed. For example, India has introduced food regulations based on the WHO South East Asia Region nutrient profile model, which defines and differentiates foods that are likely to be part of a healthy diet, vs UPFs high in sodium, free sugars, saturated fat, total fat and trans-fatty acids [43]. This profiling allows for regulations on trade to be defined and enforced, which could be an option in the Pacific context to minimise UPF intake.

Regulatory measures should be accompanied by health promotion messages about reducing the consumption of UPFs and increasing the consumption of minimally and unprocessed foods. A global systematic review suggests that dietary guidelines could discourage the consumption of UPFs by specifically referring to and defining the term ‘ultra-processed’, outlining the negative health impacts that increased UPF consumption can have, and by describing the characteristics of ultra-processing [44]. Currently, Fijian dietary guidelines encourage ‘eating less’ processed foods, and highlight the lack of nutrients in processed foods [45]. Although this is positive, UPFs could be clearly defined in the Fijian dietary guidelines, as a way to support reducing sodium and sugar intake, and for reducing the consumption of energy-dense yet nutrient-poor foods. Our findings suggest that specific population subgroups, such as younger age groups, ethnic groups and rural communities in Fiji, could be focused on in supporting to adhere to these guidelines.

### Ultra-processed foods and food security

The Pacific Island region faces food security challenges further intensified by climate change and natural disasters [17]. In Fiji, natural events such as cyclones, storms and rising sea levels may negatively impact diets [46]. Natural disasters often make food and clean water difficult to obtain and food kits from overseas are given as aid. Generally, UPFs are included in food aid kits due to their longevity, limited preparation requirements, and high carbohydrate content to provide energy [47]. For example, after Cyclone Pam in Vanuatu, food aid included imported white rice, tinned meat, and instant noodles [48]. Traditional foods can be used in food aid. For example, in disaster-prone areas in India, often cereals are given as part of food aid which are not traditionally consumed foods [49]. Instead, researchers substituted highly processed cereals with locally grown and traded pulses and millets [49]. This highlights that modifying emergency food aid kits may reduce food wastage, positively impact the environment, boost the agriculture sector [49] and are a healthier alternative to UPFs [50].

### Strengths and weaknesses

This is the first study to quantitatively assess UPF consumption in the central division of Fiji, across both a rural and urban area. Further, the Intake24 food composition database was tailored to Fiji by adapting UK and New Zealand data to include commonly consumed foods in Fiji. Overall, the assumptions made determining the categorisation of food products favoured under-estimation of UPF consumption (for example, for roti while it can be store bought with a long shelf life, it is also commonly homemade in Fiji, so all roti was classified as homemade and not ultra-processed). However, we do acknowledge that 24-h recalls do not capture discretionary use of sodium (e.g. cooking salt or table salt used at the household level) but only sodium in processed or UPFs. This potentially leads to a significant underestimation of total sodium intake. Therefore, the contribution of UPFs to total sodium intake may be significantly overestimated and discretionary salt may vary between population groups depending on the frequency of eating out and household cooking. However, our results should be interpreted in light of some limitations. A single 24-h diet recall was used. This provides self-reported intake data at a single point in time, and as such may not be reflective of habitual diets. Data was also not collected on weekend days which may change what foods were consumed. We also acknowledge that there are differing levels of processing of food items in the UK, New Zealand and Fiji, which may have impacted the data reported. There are also limitations to self-reported information such as the influence of social desirability bias which is dependent on the participant accurately remembering and reporting all foods consumed in the past 24 h [51]. For example, evidence suggests that participants tend to under-report snack foods, which are often UPFs, meaning that our convenience food findings may be an underestimation [52]. We aimed to minimise these limitations by having an interviewer administer a three-pass diet recall approach, allowing for multiple rounds of questioning, including prompts for frequently forgotten foods and drinks (for example snack foods and drinks consumed between meals). There are also limitations to the NOVA categorisation system, as the system categorises foods broadly and does not provide clear distinctions and guidelines for classifying specific foods, ingredients or mixed dishes [53]. To address this limitation, when unsure if a food was processed or UPF, we classified it as processed, potentially underestimating the association. We also followed a published approach for the classification of mixed cooked by Coyle et al. [24].

## Conclusion

This study found that UPFs are a significant contributor to energy intake along with the intake of sodium, sugar and fat in a population of adults in the Central Division of Fiji, with the main sources being bread and bakery products, non-alcoholic beverages, and convenience foods. Our findings suggest that there is an opportunity to focus regulation and consumer change campaigns in Fiji to target UPF consumption and promote the consumption of less processed options, to support healthier diets and reduce the burden of diet-related disease.

## Supplementary Information

Additional File 1: Percent contribution of NOVA categorisation to energy, sodium, sugar and fat intake by subgroups; Additional File 1: Table 1: Percent contribution of unprocessed or minimally processed foods to energy, sodium, sugar and fat intake by subgroups; Additional File 2: Table 2: Percent contribution of processed foods to energy, sodium, sugar and fat intake by subgroups.

## Data Availability

The datasets used and or analysed in this study are available from the corresponding author upon reasonable request.

## Abbreviations

UPF: Ultra-processed foods
NCDs: Non-communicable diseases
WHO: World Health Organization
GACD: Global Alliance for Chronic Diseases
SSB: Sugar-sweetened beverages
