# Supplementary material for: Ultra-processed food consumption in the central division of Fiji
Source: BMC Med. 2025 Feb 21;23:109. doi: 10.1186/s12916-025-03947-w (PMC11846173; doi:10.1186/s12916-025-03947-w)
Supplement: Supplementary file 1 — Additional File 1: Percent contribution of NOVA categorisation to energy, sodium, sugar and fat intake by subgroups; Additional File 1: Table 1: Percent contribution of unprocessed or minimally processed foods to energy, sodium, sugar and fat intake by subgroups; Additional File 2: Table 2: Percent contribution of processed foods to energy, sodium, sugar and fat intake by subgroups. [file 12916_2025_3947_MOESM1_ESM.docx]

|  | **Additional File Table 1: Percent contribution of unprocessed foods or minimally processed foods to energy, sodium, sugar and fat intake by subgroups** | | | | | | | | |
| --- | --- | --- | --- | --- | --- | --- | --- | --- | --- |
|  |  | **By sex** | | **By age group** | | **By ethnicity** | | **By area** | |
|  | **Total** | **Female** | **Male** | **18 to 44 years** | **45 years and up** | **ITaukei** | **FID and FOD** | **Deuba** | **Waidamudamu** |
| Energy | 47.6 (45.3 to 49.9) | 48.1 (45.0 to 51.3) | 47.1 (43.8 to 50.3) | 46.5 (43.6 to 49.5) | 49.5 (46.1 to 52.8) | 51.8 (48.5 to 55.1) | 43.9 (40.8 to 47.0) | 48.2 (45.0 to 51.4) | 46.8 (43.8 to 49.7) |
| Sodium | 39.1 (36.4 to 41.7) | 38.3 (34.5 to 42.0) | 39.8 (36.0 to 43.7) | 37.9 (34.4 to 41.5) | 41.0 (36.9 to 45.0) | 38.0 (33.8 to 42.2) | 39.9 (36.5 to 43.4) | 39.3 (35.5 to 43.1) | 38.6 (35.1 to 42.2) |
| Sugar | 52.9 (50.2 to 55.7) | 54.1 (50.3 to 58.0) | 51.7 (47.8 to 55.7) | 51.3 (47.6 to 55.0) | 55.7 (51.8 to 59.7) | 61.1 (57.0 to 65.2) | 45.7 (42.0 to 49.5) | 53.4 (49.5 to 57.3) | 52.2 (48.6 to 55.9) |
| Fat | 44.4 (41.7 to 47.2) | 43.2 (39.4 to 47.0) | 45.7 (41.8 to 49.7) | 43.5 (39.9 to 47.1) | 46.1 (41.9 to 50.4) | 45.4 (41.2 to 49.6)^2^ | 43.6 (40.0 to 47.2)^2^ | 44.8 (40.8 to 48.7) | 44.0 (40.4 to 47.5) |

*^2^Significant difference by ethnicity*

|  | | **Additional File Table 2: Percent contribution of processed foods to energy, sodium, sugar and fat intake by subgroups** | | | | | | | | |
| --- | --- | --- | --- | --- | --- | --- | --- | --- | --- | --- |
|  |  | | **By sex** | | **By age group** | | **By ethnicity** | | **By area** | |
|  | **Total** | | **Female** | **Male** | **18 to 44 years** | **45 years and up** | **ITaukei** | **FID and FOD** | **Deuba** | **Waidamudamu** |
| Energy | 30.9 (28.8 to 33.1) | | 30.0 (27.1 to 32.9) | 31.9 (28.8 to 35.0) | 31.1 (28.3 to 34.0) | 30.6 (27.5 to 33.7) | 21.4 (18.7 to 24.1) | 39.3 (36.1 to 42.5) | 28.5 (25.5 to 31.5) | 34.7 (31.9 to 37.5) |
| Sodium | 38.2 (35.5 to 40.8) | | 38.2 (34.4 to 41.9) | 38.1 (34.3 to 42.0) | 39.2 (35.6 to 42.7) | 36.4 (32.5 to 40.3) | 31.2 (27.1 to 35.2)^2^ | 44.3 (40.7 to 47.8)^2^ | 36.1 (32.3 to 39.9) | 41.2 (37.7 to 44.7) |
| Sugar | 23.1 (20.9 to 25.2) | | 22.1 (19.1 to 25.0) | 24.1 (20.9 to 27.3) | 22.3 (19.4 to 25.1) | 24.5 (21.3 to 27.7) | 14.2 (11.6 to 16.8) | 30.9 (27.5 to 34.2) | 21.8 (18.8 to 24.9) | 24.9 (22.1 to 27.8) |
| Fat | 37.0 (34.3 to 39.6) | | 37.4 (33.6 to 41.2) | 36.5 (32.9 to 40.2) | 37.9 (34.4 to 41.4) | 35.4 (31.5 to 39.3) | 30.3 (26.3 to 34.2)^2^ | 42.8 (39.3 to 46.4) ^2^ | 34.6 (30.9 to 38.4) | 40.5 (37.1 to 44.0) |

*^2^Significant difference by ethnicity*
